# The human oral cavity microbiota composition during acute tonsillitis: a cross-sectional survey

**DOI:** 10.1186/s12903-019-0956-5

**Published:** 2019-12-05

**Authors:** Yun Kit Yeoh, Man Hin Chan, Zigui Chen, Eddy W. H. Lam, Po Yee Wong, Chi Man Ngai, Paul K. S. Chan, Mamie Hui

**Affiliations:** 1 Centre for Gut Microbiota Research, Faculty of Medicine, The Chinese University of Hong Kong, Hong Kong SAR, China; 2 Department of Microbiology, Faculty of Medicine, The Chinese University of Hong Kong, Hong Kong SAR, China; 3 Li Ka Shing Institute of Health Sciences, Faculty of Medicine, The Chinese University of Hong Kong, Hong Kong SAR, China; 4 Department of Otorhinolaryngology, Head and Neck Surgery, Yan Chai Hospital, Hong Kong SAR, China

**Keywords:** Mouth rinse, 16S ribosomal RNA gene, Microbial community, Prevotella, Smoking, Fusobacteria

## Abstract

**Background:**

Microbial culture-based investigations of inflamed tonsil tissues have previously indicated enrichment of several microorganisms such as *Streptococcus*, *Staphylococcus* and *Prevotella*. These taxa were also largely reflected in DNA sequencing studies performed using tissue material. In comparison, less is known about the response of the overall oral cavity microbiota to acute tonsillitis despite their role in human health and evidence showing that their compositions are correlated with diseases such as oral cancers. In addition, the influence of subject-specific circumstances including consumption of prescription antibiotics and smoking habits on the microbiology of acute tonsillitis is unknown.

**Methods:**

We collected oral rinse samples from 43 individuals admitted into hospital for acute tonsillitis and 165 non-disease volunteers recruited from the public, and compared their microbial community compositions using 16S rRNA gene sequencing. We assessed the impact of tonsillitis, whether subjects were prescribed antibiotics, the presence of oral abscesses and their smoking habits on community composition, and identified specific microbial taxa associated with tonsillitis and smoking.

**Results:**

Oral rinse community composition was primarily associated with disease state (tonsillitis vs non-tonsillitis) although its effect was subtle, followed by smoking habit. Multiple *Prevotella* taxa were enriched in tonsillitis subjects compared to the non-tonsillitis cohort, whereas the non-tonsillitis cohort primarily showed associations with several *Neisseria* sequence variants. The presence of oral abscesses did not significantly influence community composition. Antibiotics were prescribed to a subset of individuals in the tonsillitis cohort but we did not observe differences in community composition associated with antibiotics consumption. In both tonsillitis and non-tonsillitis cohorts, smoking habit was associated with enrichment of several *Fusobacterium* variants.

**Conclusions:**

These findings show that the oral cavity microbial community is altered during acute tonsillitis, with a consistent enrichment of *Prevotella* during tonsillitis raising the possibility of targeted interventions. It also supports the possible link between smoking, *Fusobacteria* and oral cancers.

## Background

The human oral microbiome has been relatively well characterized [1–4], and ongoing efforts are focused on studying compositional and spatial organization of oral communities as well as similarities and differences across populations [5]. Another widely appreciated aspect is the role of oral microbial communities in oral and systemic diseases [6, 7]. One common condition- the inflammation of tonsils (termed tonsillitis), is also regarded to have a microbial aetiology. Tonsils are part of the lymphatic system and can become inflamed when infected by bacteria or viruses. While tonsillitis is usually a self-limiting disease, abscesses can form in some cases and require surgical intervention to drain accumulated pus. Culture-based characterization of microorganisms in tonsil tissues of patients with tonsillitis have reported a polymicrobial association commonly involving group A streptococci, *Staphylococcus aureus*, *Streptococcus pneumoniae*, *Haemophilus influenzae*, and members of the *Prevotella*, *Bacteroides*, *Fusobacterium*, *Porphyromonas* and *Veillonella* genera [8–10]. Since many human-associated microorganisms are not cultivable under laboratory conditions, recent studies have applied culture-independent DNA sequencing methods to identify the kinds of microorganisms present and quantify their abundances in the human microbiome [11]. By sequencing the microbial small subunit ribosomal RNA gene (16S), a study of microorganisms enriched in tonsillar tissue from adult subjects with recurrent tonsillitis identified enrichment of members of the *Treponema*, *Fusobacterium*, *Streptococcus*, *Selenomonas*, *Gemella*, *Tannerella* and *Prevotella* genera compared to healthy individuals [12].

While it has been reported that tonsil tissue-associated microbial communities are altered in subjects with tonsillitis, there are no studies describing whether the total oral cavity microbiome is also influenced by this condition. The oral cavity microbiome is commonly studied by collecting oral rinse samples [11, 13, 14], and its composition has been shown to correlate with diseases such as oral cavity and oropharyngeal cancers [13, 15]. Here, we collected oral rinse samples from patients showing symptoms of acute tonsillitis and compared their oral cavity microbial community composition to healthy individuals without oral disease. We hypothesized that the oral microbiome composition in tonsillitis patients differed from healthy individuals, and that disease state (i.e. tonsillitis vs no disease) was the primary factor attributable to differences in community composition. We also investigated the influence of prescription antibiotics, the presence of oral abscesses and smoking habits on oral rinse community compositions.

## Methods

### Subjects and sample collection

Individuals admitted to the Department of Otorhinolaryngology, Head and Neck Surgery, Yan Chai Hospital, Hong Kong SAR for symptoms of acute tonsillitis were recruited for this observational study. No interventions were made based on the needs or findings of this study. Firstly, demographic (such as age, gender, smoking habits) and medical information (such as antibiotics prescribed prior to admission) were collected. Disease severity was noted based on whether subjects required incision and drainage of abscesses. Before any treatment or medication was prescribed, subjects first rinsed their mouths with drinking water to remove any food debris. They then swilled 10 ml of sterile 0.9% saline solution in their oral cavities for 1 min, and collected the saline rinse in sterile 70 ml specimen jars. A separate cohort of healthy volunteers was recruited from the public to provide oral rinse samples using the same collection method to serve as a reference for the oral microbiome in the local population. Demographic information was recorded through a self-administered validated questionnaire (Additional file 2). All oral rinse samples were stored at − 20 °C until DNA extraction.

### DNA extraction and 16S amplicon sequencing

DNA extraction and 16S amplicon sequencing were performed as previously described [16]. Briefly, DNA was extracted from 1 ml of oral rinse using the QIAGEN DNeasy PowerSoil Kit according to manufacturer’s instructions, and the V3 and V4 variable regions of the 16S gene were amplified by polymerase chain reaction (PCR). DNA-free negative and mock microbial community positive controls (ZymoBIOMICS Microbial Community DNA Standard, catalogue number D6305) were included in the PCR. Following amplification, sequencing adapters and multiplex indices were added to the PCR products in a second PCR and the resulting amplicons purified using the QIAquick Gel Extraction Kit. Purified final products were sequenced on an Illumina MiSeq (2 × 300 bp) using the v3 MiSeq Reagent Kit.

### 16S sequence data processing

Demultiplexed raw sequence data were imported into QIIME 2 v2018.6 [17]. Using the DADA2 workflow in QIIME 2, primer and low quality sequences were trimmed, and remaining reads subsequently denoised and merged. Alpha diversity metrics (species richness, Shannon index and Faith’s phylogenetic diversity) were calculated based on sequence counts normalised to a depth of 4000 sequences per sample. To assign taxonomy to sequences, a classifier was first trained on reference 16S sequences extracted from the SILVA 16S database release 128 [18] using the 16S gene V3–4 universal primer sequences. This classifier was then run on representative sequences produced by DADA2 to assign probable taxonomies to the corresponding sequences. The final non-normalized counts table based on exact sequence variants (ESVs) and their 16S taxonomies, as well as UniFrac distances of community composition were exported from QIIME 2, and used as input in R for statistical analysis.

### 16S amplicon-based microbial community composition analyses

The resulting ESV counts table from QIIME 2 was imported together with sample metadata into R v3.5.1. A centered log-ratio transformation was applied to ESV counts before downstream analyses to ensure that the counts fulfilled assumptions of independence between predictor variables for statistical analyses (explained in [19]). Permutational multivariate analysis of variance (PERMANOVA) was used to assess whether factors such as disease state (tonsillitis vs healthy), smoking habit, age and gender significantly influenced community composition, as well as to determine the amount of variation in community composition attributable to each of these factors. Principal component analysis (PCA) and principal coordinate analysis (PCoA) ordinations were used to visualise the clustering of samples based on their compositional similarities. Association of ESVs to experimental factors were identified using the linear discriminant analysis effect size (LEfSe) algorithm [20] implemented in the Huttenhower Lab Galaxy web application framework available online (http://huttenhower.sph.harvard.edu/galaxy/), and also with a random forest classifier. PERMANOVA, PCA and PCoA are implemented in the vegan R package v2.4–6, power calculations in the HMP R package [21], and random forest in the randomForest R package v4.6–14. Figures were edited in Inkscape v0.92 for clarity.

## Results

### Oral rinse microbiome composition of healthy individuals and subjects with tonsillitis

A total of 43 subjects with acute tonsillitis were recruited to assess the oral rinse microbial community during tonsillitis. Of these subjects, 15 were prescribed antibiotics prior to admission, and 20 required incision and drainage of abscesses (Table 1). Their mean white cell counts measured as per hospital admissions procedure was 15.1 × 10^9^ cells/μl blood (median 13.9 × 10^9^ cells/μl), of which 76.9% on average was comprised of neutrophils suggesting a bacterial aetiology. As a reference non-tonsillitis cohort, 165 healthy volunteers were recruited from the public. These healthy subjects reported no history of chronic diseases or chronic use of medication and antibiotics in the past year. Sequencing of PCR amplicons from these 208 oral rinse samples produced 3,162,740 sequences, with a median of 14,909 sequences per sample after quality filtering and merging read pairs. With this design and sequencing depth, the study had 85.3% power to detect differences in community composition between tonsillitis and healthy subjects (significance level = 0.05, number of reads per sample = 14,000).

**Table 1:** Characteristics of the study subjects

Characteristic	Healthy cohort (*N* = 165)	Tonsillitis cohort (*N* = 43)
Male: female ratio	2.2	2.3
Median age (interquartile range)	40 (31) years	36 (23.5) years
Antibiotics (prior to sampling)	none	In 15 subjects
Abscesses (requiring incision and drainage)	none	In 20 subjects
Smokers	17 former, 11 current, 137 non	2 former, 18 current, 23 non

**Fig. 1:**
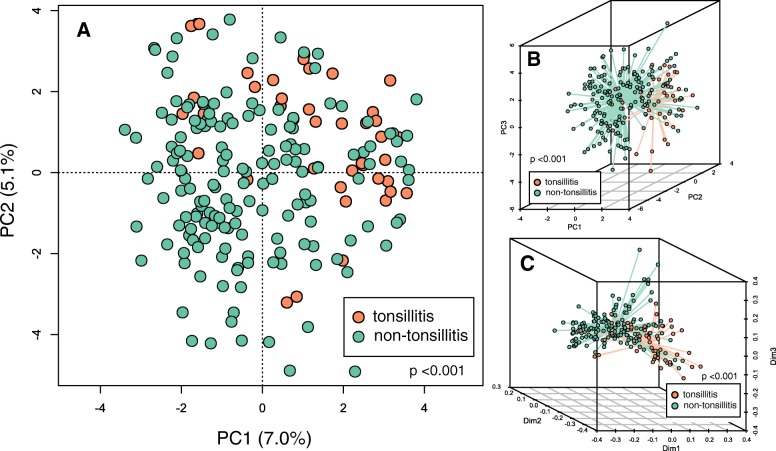
Ordination of oral rinse microbial communities in healthy individuals and patients with tonsillitis. **a** Principal component analysis (PCA) of centered log ratio-transformed exact sequence variant (ESV) counts. Each circle represents community composition of one sample; the closer two dots are the more compositionally similar their microbial communities. **b** Three dimensional representation of PCA in panel A, with spokes connecting samples to their respective centroids according to cohort (healthy or tonsillitis). **c** Principal coordinate analysis (PCoA) of weighted UniFrac distances calculated in QIIME 2. Green circles represent samples from non-tonsillitis individuals, orange circles represent samples from patients with tonsillitis. Community composition of samples from tonsillitis patients and healthy individuals significantly differed irrespective of metric used (transformed ESV counts and weighted UniFrac) (*p* < 0.001, PERMANOVA)

To determine whether oral microbial community composition significantly differed between tonsillitis patients and healthy subjects, we applied a centered log-ratio transformation on 16S counts data and then performed a PCA. Similarly, we also performed a PCoA on weighted UniFrac distances that account for relatedness among members of the community. In both ordinations, samples primarily clustered according to disease state (i.e. tonsillitis vs healthy cohorts) (Fig. 1) (*p* < 0.001, permutation test), indicating that the oral rinse microbiome composition was significantly altered in patients with tonsillitis relative to healthy individuals. These observations were supported by a PERMANOVA (stratified by cohort) indicating that cohort (i.e. tonsillitis vs healthy), smoking habit (current, former and non-smokers), age and gender were significantly associated with microbial community composition in descending size of effect (Table 2). Alpha diversity, however, did not differ between the tonsillitis and healthy cohorts (Additional file 1: Figure S1) (*p* > 0.05, Kruskal-Wallis test).

**Table 2:** PERMANOVA of oral microbial community composition in healthy and tonsillitis cohorts

	Sums of squares	Mean squares	F model	*R*^2^	*p*-value
Disease state	8485	8484.6	4.032	0.019	< 0.001
Smoking	7360	3680.0	1.749	0.016	< 0.001
Age	3743	3743.4	1.779	0.008	< 0.001
Gender	3015	3014.8	1.433	0.007	0.0110
Antibiotics	2236	2236.4	1.063	0.005	0.3263
Residuals	423,001	2104.5		0.945	

### Microbial taxa associated with healthy individuals and subjects with tonsillitis

**Fig. 2:**
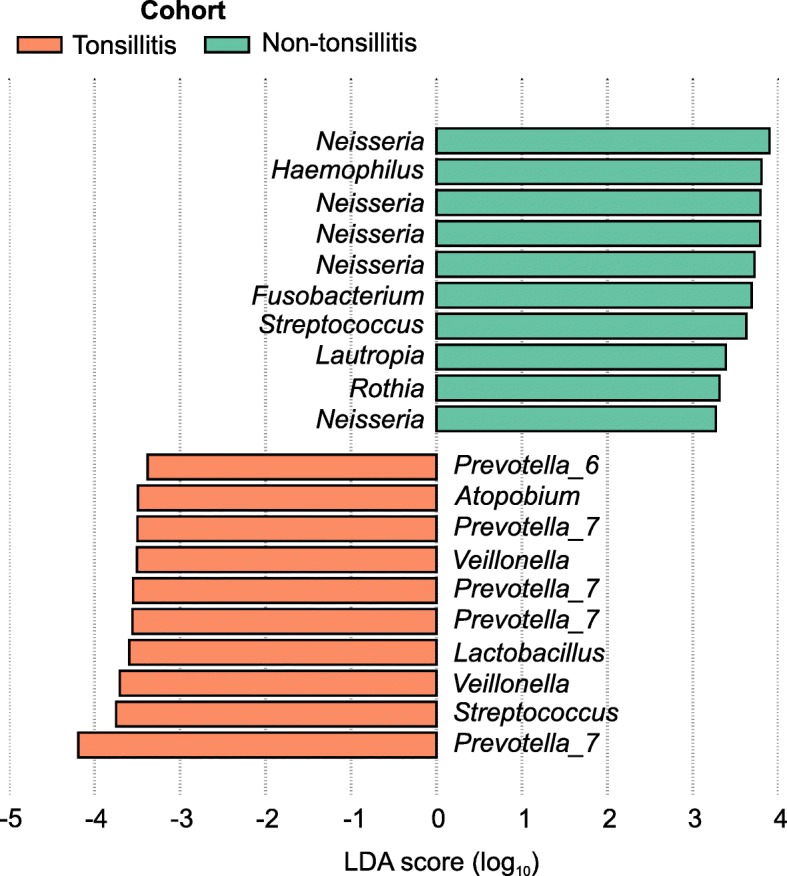
Top 10 exact sequence variants (ESVs) associated with the tonsillitis and healthy cohorts ranked according to linear discriminant analysis (LDA) effect size. ESVs were identified using the LDA effect size (LEfSe) algorithm with default parameters. Taxonomy labels indicate genus level classification (or lowest rank available) with reference to the SILVA database. Green bars represent ESVs associated with non-tonsillitis individuals, orange bars represent ESVs associated with patients with tonsillitis

As there was no interaction between tonsillitis disease status with smoking habit, age or gender indicated by PERMANOVA, we directly compared community composition between the tonsillitis and non-tonsillitis cohorts to identify differentially enriched taxa. Using the linear discriminant analysis (LDA) effect size (LEfSe) algorithm, 103 ESVs were significantly associated with the tonsillitis cohort whereas 57 were significantly associated with the healthy cohort (*p* < 0.05, LDA score > 2). Five of the top 10 ESVs associated with the tonsillitis cohort were classified as *Prevotella*, and the remaining were a *Streptococcus*, two *Veillonella*, a *Lactobacillus* and an *Atopobium* (Fig. 2). In contrast, the oral rinse community of the healthy cohort was associated with *Neisseria*, *Haemophilus*, *Fusobacterium*, *Streptococcus*, *Lautropia* and a *Rothia*. These findings were consistent with those generated using a random forest classifier to compare community composition between tonsillitis subjects and healthy individuals, in which *Atopobium*, *Veillonella* and *Prevotella* were implicated in tonsillitis samples (Additional file 1: Figure S2).

### Secondary factors associated with oral microbiota composition in subjects with tonsillitis

**Fig. 3:**
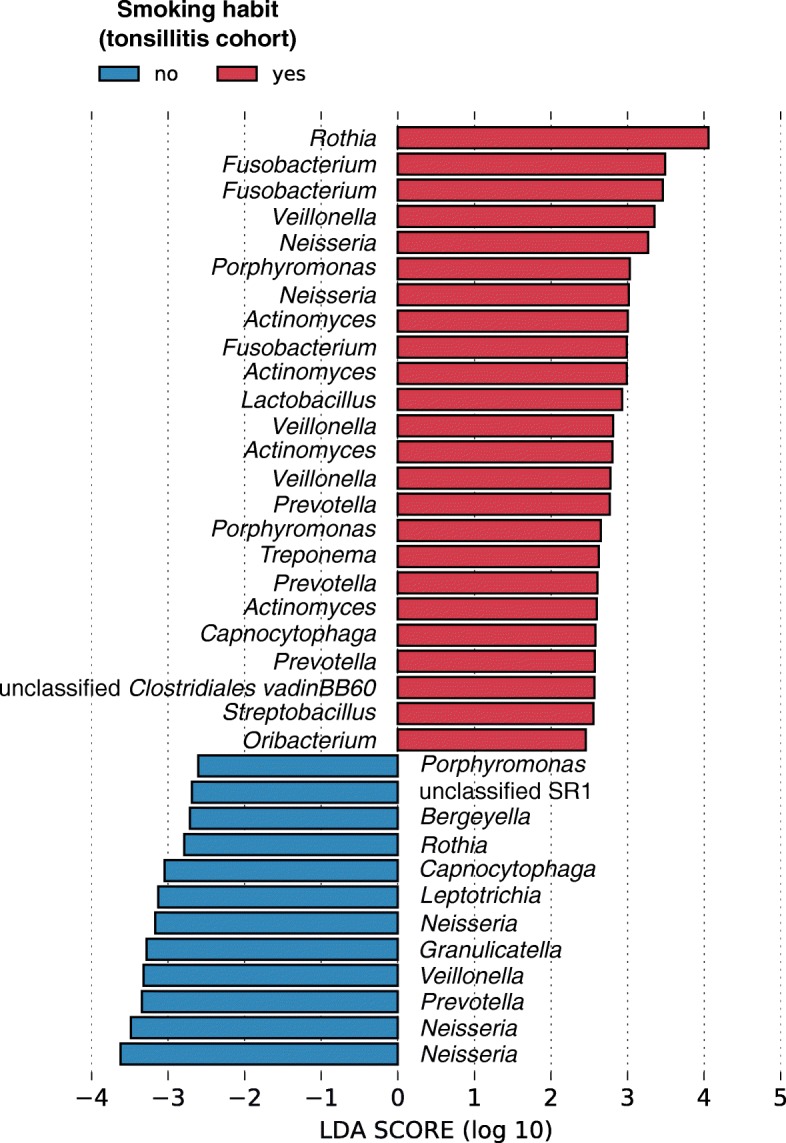
Exact sequence variants (ESVs) associated with smokers and non-smokers in the tonsillitis cohorts ranked according to linear discriminant analysis (LDA) effect size. ESVs were identified using the linear discriminant analysis effect size (LEfSe) algorithm. Only ESVs with an LDA score of at least 2 in their respective groups are shown. Genus level classification (or lowest rank available) with reference to the SILVA database is provided. Red bars represent ESVs associated with smokers, whereas blue bars represent ESVs associated with non-smokers

Prior to recruitment into this study, 15 subjects from the tonsillitis cohort were prescribed antibiotics by general practitioners for an average duration of 3.1 days (standard deviation of 2.5 days). Amoxicillin/clavulanate was prescribed to nine subjects, and amoxicillin, azithromycin, cefuroxime and levofloxacin to one subject each. Two subjects were uncertain of their prescribed antibiotics. In addition, 20 subjects underwent incision and drainage of abscesses, suggesting that they experienced a more severe bout of disease. Since the consumption of antibiotics and disease severity are known to influence microbial community composition, we analysed data from the tonsillitis cohort alone to assess whether prescription of antibiotics and presence of abscesses requiring surgical intervention were associated with oral rinse community composition. A PERMANOVA indicated that community composition was not associated with antibiotics consumption, the presence of abscesses, age or gender, but identified a significant difference between smokers and non-smokers (Additional file 1: Table S1). These differences were attributed to associations of ESVs such as *Rothia*, *Fusobacterium* and *Actinomyces* with smokers, and multiple sequence variants of *Neisseria*, *Veillonella*, *Prevotella* and *Granulicatella* with non-smokers although ESVs classified under the same genera were also detected in smokers (Fig. 3) (LEfSe; two former smokers were excluded). Differences in oral community composition were also detected in the healthy cohort in which smoking status, age and gender were significantly associated in descending size of effect (Additional file 1: Table S2). Three fusobacterial ESVs were implicated in the oral rinse microbial communities in smokers and former-smokers from the healthy cohort, although another two were associated with non-smokers (Additional file 1: Figure S3). A *Neisseria* ESV was again the most strongly associated taxa in non-smokers. These results suggest that members of the *Fusobacterium* genus are enriched in the oral cavities of smokers, however, further validation is required to identify these taxa beyond the genus level resolution provided by conventional 16S rRNA gene sequencing.

## Discussion

Tonsillitis is traditionally associated with an overgrowth of bacterial taxa such as *Bacteroides*, *Fusobacterium*, *Veillonella*, *Prevotella*, *Streptococcus*, *Staphylococcus* and *Haemophilus* based on laboratory cultures of tonsillar tissue and material from abscesses [8, 22]. In addition to supporting these observations, culture-independent microbial community surveys based on DNA sequencing have also uncovered additional associations with other taxa such as *Treponema*, *Fusobacterium, Gemella* and *Tannerella* [12]. Our data extended these findings to a cohort of tonsillitis patients in Hong Kong in which we observed a subtle but significant difference in oral rinse microbial community composition compared to healthy individuals, in line with previous reports that composition of microbial communities in oral rinse samples is predictive of oral disease [13]. An even smaller effect of gender and age was identified, although these two factors were only significantly associated with oral community composition in healthy subjects when the tonsillitis and healthy cohorts were analysed separately. We postulate that the lack of association in tonsillitis subjects could be due to a larger influence of tonsillitis overriding effects linked to age and/or gender, and also partly because of reduced power in detecting age/gender differences in community composition in the smaller tonsillitis cohort compared to healthy individuals (Table 1).

Although compositional differences in oral community composition linked to tonsillitis were subtle as indicated by PERMANOVA and the lack of statistical difference in alpha diversity indexes between cohorts, five of the top 10 ESVs associated with tonsillitis consistently classified as *Prevotella* suggested that multiple members of this genus may be involved with oral disease. Indeed, *Prevotella* sequence variants have been implicated in various oral conditions such as periodontitis [23–25] and endodontic abscesses [26], and are possibly enriched through their preferential utilization of proteins and production of cytotoxic end products [27, 28]. We also identified several *Streptococcus*, *Veillonella*, *Lactobacillus* and *Atopobium* variants associated with the tonsillitis cohort. While these tonsillitis-associated taxa were mostly distinct from those associated with healthy samples, *Streptococcus* ESVs were linked to both tonsillitis and healthy cohorts (Fig. 2). A limitation of using 16S amplicon sequencing is the insufficient resolution in classifying species level differences [29, 30], which is compounded by microbial genera in oral communities encompassing multiple species and/or polyphyly in their phylogenetic classifications [3, 31]. Ideally, full-length 16S sequences should be obtained to confirm exact species identities of any implicated microorganisms.

While the main aim of this study was to describe differences in oral community composition between patients with tonsillitis and healthy individuals, we also observed secondary differences in community composition linked to smoking in both healthy and tonsillitis cohorts. Firstly, we wish to point out that smokers and former smokers were underrepresented in numbers compared to non-smokers (29 smokers, 19 former smokers and 160 non-smokers in total). Nevertheless, we identified associations of multiple *Fusobacterium* ESVs with smokers and *Neisseria* with non-smokers in both the tonsillitis (Fig. 3) and healthy cohorts (Additional file 1: Figure S3) consistent with other studies comparing oral community composition between smokers and non-smokers. Previous oral community surveys have consistently reported reductions of *Neisseria* detected in mucosal surfaces [32], oropharynx [33] and oral rinses [34–36] of smokers compared to non-smokers. Conversely, taxa such as *Veillonella*, *Actinomyces* and *Fusobacterium* have been found to be increased in the oral cavity of smokers compared to non-smokers, although contrasting results exist [33–36] possibly due to differences in biogeography of samples and technical limitations of the 16S gene in delineating microbial species (as mentioned above). In addition, we identified association of a *Bergeyella* and a member of candidate phylum SR1 with non-smokers, which was also found in non-smokers from a New York City cohort [37]. One notion of how smoking influences the oral microbial community composition is that cigarette smoke creates anaerobic conditions favouring anaerobic microorganisms such as *Veillonella*, *Fusobacterium* and *Actinomyces*, and suppresses aerobes such as *Neisseria* [32, 35, 37]. This inference is consistent with our data showing associations of other anaerobic or microaerophilic taxa such as *Fusobacterium*, *Leptotrichia*, *Porphyromonas*, *Lactobacillus* and *Treponema* in smokers from the tonsillitis and healthy cohorts, although whether they are linked to a predisposition to disease in smokers remains an open question. Most notably, *Fusobacterium*, *Leptotrichia* and *Rothia* in the oral cavity have been associated with squamous cell carcinoma [38] and oral leukoplakia [39], and thus their association with smokers suggests that smoking habits may exacerbate oral cancers through the enrichment and/or activity of these microorganisms. However, as mentioned earlier, 16S amplicon sequencing does not provide sufficient resolution in identifying species level associations. Additional studies are required to validate whether the microbial taxa implicated here indeed have oncogenic potential, and play a role in the link between smoking, microorganisms and oral cancer [40].

Although it is widely accepted that antibiotics exert a strong influence on the composition of human microbiomes, we detected no statistical difference in the oral rinse communities of patients provided with systemic antibiotics compared to those without. We wish to clarify that we likely saw no measurable effects of antibiotics consumption on the oral microbiome in this cohort because the use of antibiotics among subjects was highly variable (15 of 43 subjects received antibiotics; several classes of antibiotics) and not controlled as these were prescribed by general practitioners prior to subjects’ admission into the hospital. Therefore, findings relating to the consumption of antibiotics should be interpreted with caution.

## Conclusion

In conclusion, we found that the oral rinse microbial community composition is altered during tonsillitis compared to healthy individuals. Specifically, there was an enrichment of *Prevotella* taxa in the tonsillitis cohort, raising the possibility of using targeted antimicrobial interventions to treat acute tonsillitis by eradicating *Prevotella* species. We also identified a link with smoking habits independent of tonsillitis; several fusobacterial taxa were enriched in smokers compared to non-smokers, further supporting a microbial link between smoking and oral cancers. The nature of these fusobacterial taxa as well as their associations with smoking and oral cancers should be investigated in more detail.

## Supplementary information

**Additional file 1: Figure S1.** Alpha diversity of oral rinse microbial communities in healthy individuals and patients with tonsillitis. **Figure S2.** Top 10 genera discriminating oral rinse microbial communities between tonsillitis and non-tonsillitis cohorts. **Figure S3.** Top exact sequence variants associated with smokers and former smokers vs non-smokers in the non-tonsillitis cohort ranked according to effect size. **Table S1.** PERMANOVA of oral microbial community composition in tonsillitis cohort. **Table S2.** PERMANOVA of oral microbial community composition in non-tonsillitis cohort

**Additional file 2.** Demographics questionnaire

## Data Availability

Raw sequence data generated for this study are available in the Sequence Read Archive under BioProject accession PRJNA559766. Patient data are available from the corresponding author on reasonable request.

## Abbreviations

DNA: Deoxyribonucleic acid
dNTP: deoxynucleoside triphosphate
ESV: Exact sequence variants
LEfSe: Linear discriminant analysis effect size
PCA: Principal component analysis
PCoA: Principal coordinate analysis
PCR: Polymerase chain reaction
PERMANOVA: Permutational multivariate analysis of variance
rRNA: Ribosomal ribonucleic acid
