# Supplementary material for: The human oral cavity microbiota composition during acute tonsillitis: a cross-sectional survey
Source: BMC Oral Health. 2019 Dec 5;19:275. doi: 10.1186/s12903-019-0956-5 (PMC6896734; doi:10.1186/s12903-019-0956-5)
Supplement: Supplementary file 1 — Additional file 1: Figure S1. Alpha diversity of oral rinse microbial communities in healthy individuals and patients with tonsillitis. Figure S2. Top 10 genera discriminating oral rinse microbial communities between tonsillitis and non-tonsillitis cohorts. Figure S3. Top exact sequence variants associated with smokers and former smokers vs non-smokers in the non-tonsillitis cohort ranked according to effect size. Table S1. PERMANOVA of oral microbial community composition in tonsillitis cohort. Table S2. PERMANOVA of oral microbial community composition in non-tonsillitis cohort [file 12903_2019_956_MOESM1_ESM.docx]

**The human oral cavity microbiota composition during acute tonsillitis: a cross-sectional survey**

Yun Kit Yeoh, Man Hin Chan, Zigui Chen, Eddy W. H. Lam, Po Yee Wong, Chi Man Ngai, Paul K. S. Chan, Mamie Hui


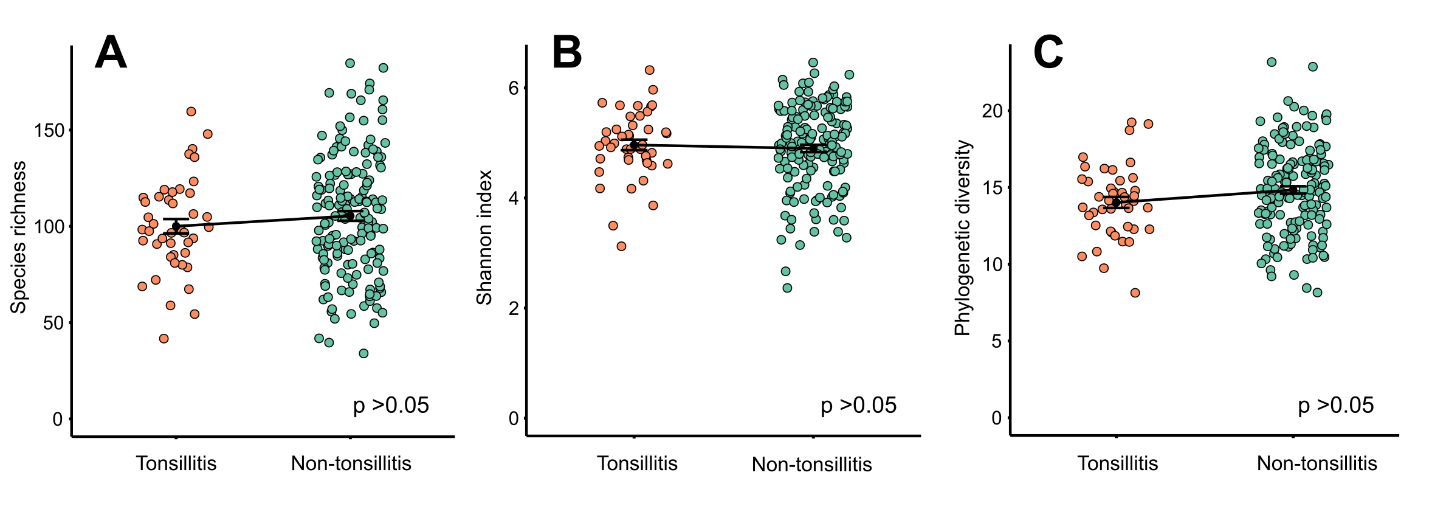


**Figure S1**. Alpha diversity of oral rinse microbial communities in healthy individuals and patients with tonsillitis. (**A**) Species richness, (**B**) Shannon index, and (**C**) Faith’s phylogenetic diversity. Values were calculated using QIIME 2 based on a normalized depth of 4000 reads per sample (one sample from the healthy cohort with 3677 reads was excluded). Black dots and error bars represent mean ± standard error. Green circles represent samples from non-tonsillitis individuals, orange circles represent samples from patients with tonsillitis. All three indexes showed no statistical difference between the tonsillitis and healthy cohorts (p >0.05, Mann-Whitney test).


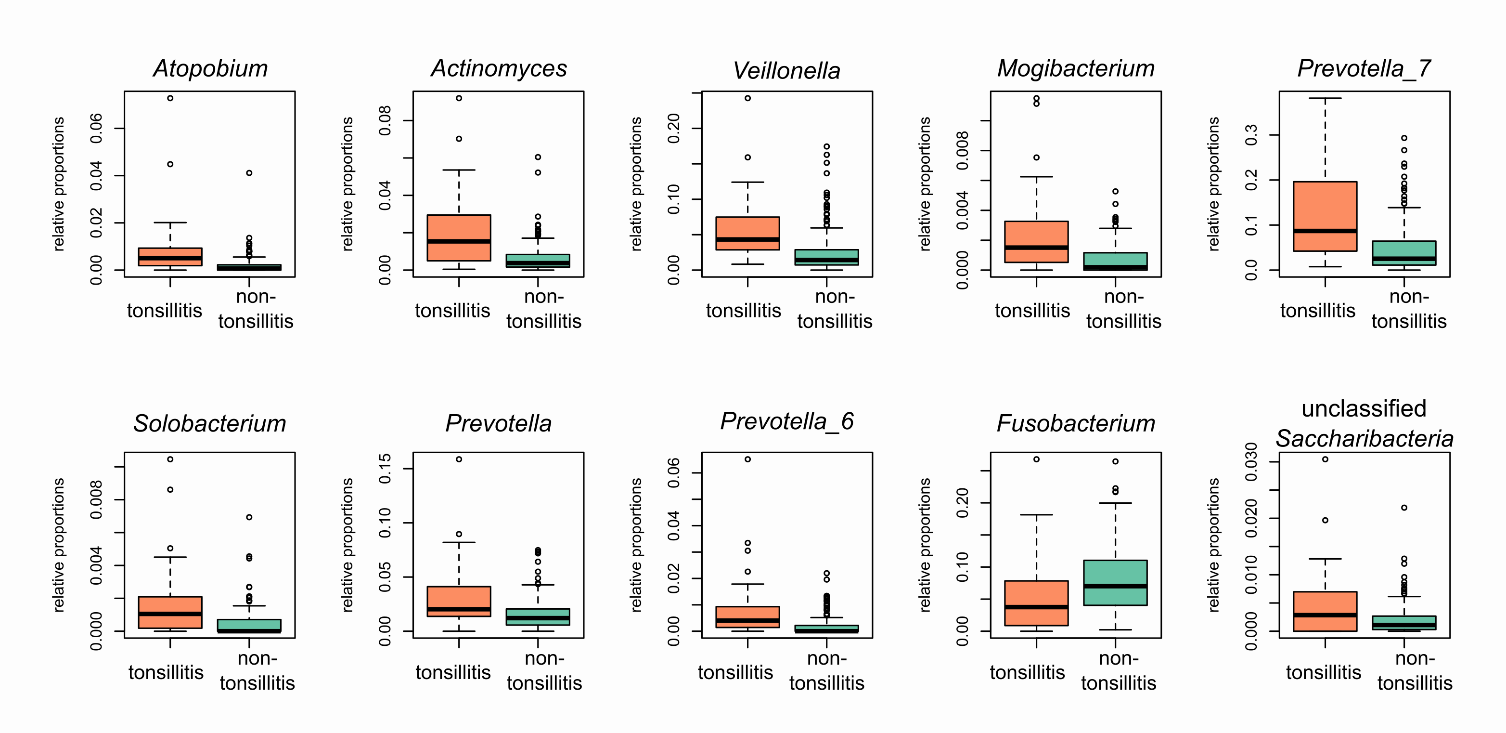


**Figure S2**. Top 10 genera (from left to right) discriminating oral rinse microbial communities between tonsillitis and non-tonsillitis cohorts. These genera were identified using a random forest classifier on centered log ratio-transformed 16S read counts collapsed at the genera level according to SILVA taxonomy. The thick centre lines within each boxplot rectangle represents median values, and the two ends of the rectangle represent upper and lower quartiles, respectively. The upper whisker extends to the highest value within 1.5x the interquartile range above the upper quartile, whereas the lower whisker extends to the lowest value within 1.5x the interquartile range below the lower quartile. Outlier values are represented as white circles. Relative proportion of total community =1. Note the different orders of magnitude in vertical axes among panels.


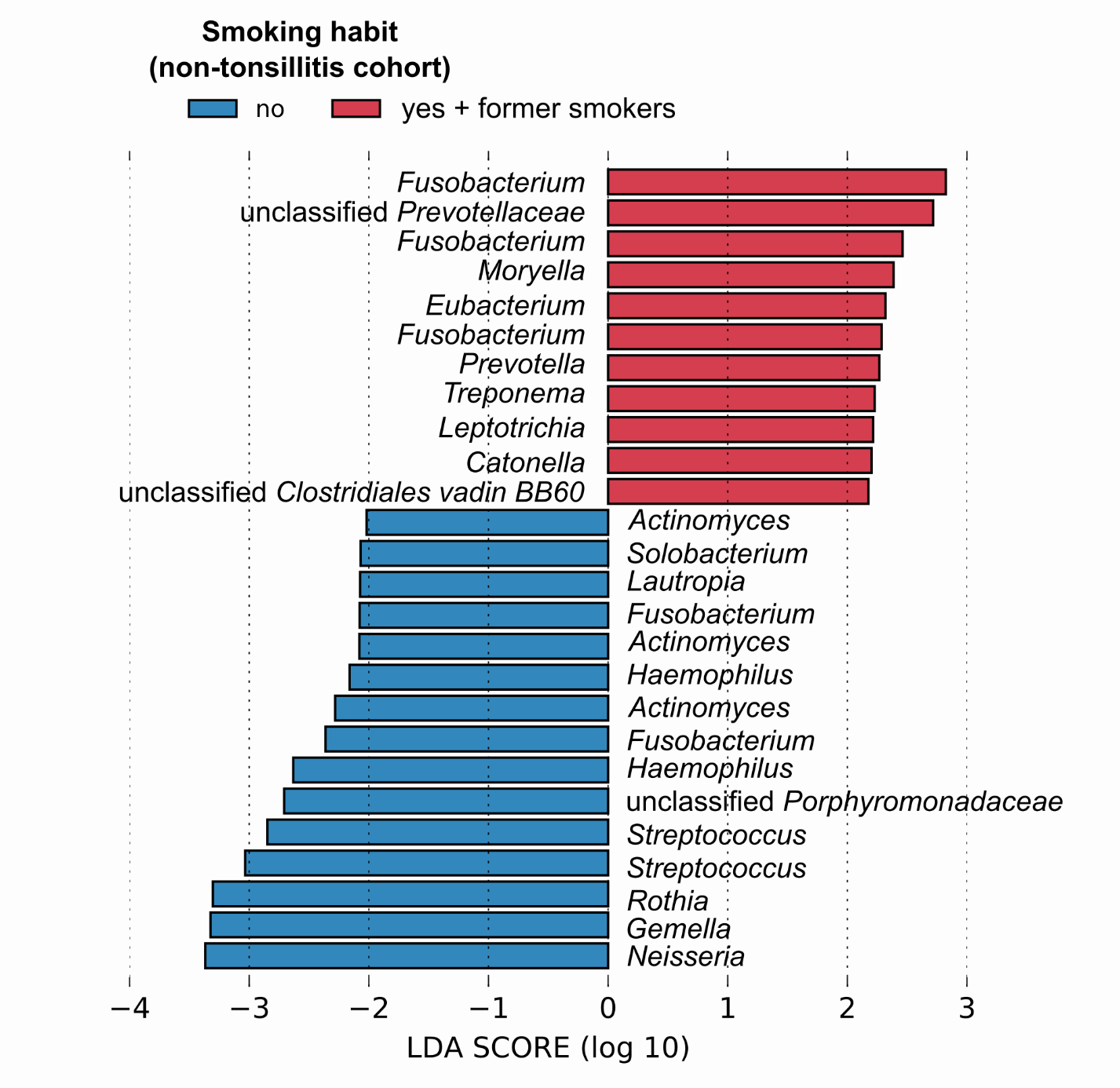


**Figure S3.** Top exact sequence variants (ESVs) associated with smokers and former smokers vs non-smokers in the non-tonsillitis cohort ranked according to effect size. ESVs were identified using the linear discriminant analysis (LDA) effect size (LEfSe) algorithm implemented in the Huttenhower Lab Galaxy web application framework. ESVs shown here are those significantly associated with their corresponding cohorts and have LDA scores ≥2 as per the default cut-off. Labels indicate genus classification (or lowest rank available) of each ESV according to SILVA taxonomy.

**Table S1.** PERMANOVA of oral microbial community composition in tonsillitis cohort

|  | Sums of squares | Mean  squares | F model | R^2^ | p-value |
| --- | --- | --- | --- | --- | --- |
| Gender | 2154 | 2154.1 | 0.997 | 0.025 | 0.437 |
| Antibiotics | 2202 | 2201.8 | 1.019 | 0.025 | 0.365 |
| Age | 2487 | 2487.3 | 1.151 | 0.028 | 0.137 |
| Smoking habit | 2984 | 2984.1 | 1.381 | 0.034 | **0.021** |
| Antibiotics : smoking habit | 2263 | 2262.6 | 1.047 | 0.026 | 0.304 |
| Residuals | 77890 | 2163.6 |  | 0.888 |  |

**Table S2.** PERMANOVA of oral microbial community composition in non-tonsillitis cohort

| Healthy cohort | SumsOfSqs | MeanSqs | F.Model | R^2^ | p-value |
| --- | --- | --- | --- | --- | --- |
| Gender | 2847 | 2847 | 1.365 | 0.008 | 0.022 |
| Age | 3835 | 3834.9 | 1.839 | 0.011 | **<0.001** |
| Smoking habit | 5907 | 2953.5 | 1.416 | 0.017 | **0.002** |
| Residuals | 327473 | 2085.8 |  | 0.944 |  |
