# Supplementary material for: The human oral cavity microbiota composition during acute tonsillitis: a cross-sectional survey
Source: BMC Oral Health. 2019 Dec 5;19:275. doi: 10.1186/s12903-019-0956-5 (PMC6896734; doi:10.1186/s12903-019-0956-5)
Supplement: Supplementary file 2 — Additional file 2. Demographics questionnaire [file 12903_2019_956_MOESM2_ESM.pdf]

研究編號: GHQ-| | | | |

填寫日期: | | | | | (年)| | | (月)| | | (日)

健康狀況調查

感謝您參與我們的研究,

你所提供的資料有助我們加深了解人類健康。

填寫本問卷須要大約 30 分鐘。

你所提供的資料將會絕對保密,

並只作研究用途。

中文大學醫學院微生物學系

A. 個人資料

A1. 在港居住 ☐☐ 年

A2. 性別: ☐[1] 男 ☐[2] 女

A3. 族裔:

☐[1] 華人(☐ 漢族/ ☐ 其他:______________)

↳ 請註明籍貫: ______________

☐[2] 非華人

A4. a. 出生日期: ☐☐☐☐(年)/☐☐(月)/☐☐(日)

b. 出生方式: ☐[1] 開刀 ☐[2] 順產(自然分娩) ☐[3] 不清楚

c. 出生後首三個月，主要餵哺方式:

☐[1] 母乳 ☐[2] 嬰兒配方奶粉 ☐[3] 不清楚

A5. 婚姻狀態:

☐[1] 未婚 ☐[2] 已婚 ☐[3] 分居

☐[4] 離婚 ☐[5] 同居 ☐[6] 喪偶

A6. 教育經歷:

☐[1] 小學或以下 ☐[2] 初中 ☐[3] 高中

☐[4] 預科或文憑 ☐[5] 大專/大學或以上

A7. 工作狀態:

☐[1] 學生 ☐[2] 待業 ☐[3] 家庭主婦 ☐[4] 退休

☐[5] 全職 ☐[6] 兼職

A8. 請列出您所從事過的職業 (我們並不需要一個完整的簡歷，而是你工作大概類型)

大概時間(例如 20-25 歲)	工作性質(例如教師、農民)

A9. 現時一同居住的人數(不包括自己)[1]: __________ 人，請註明:

[2]祖父母:______名 [3]父母:______名 [4]兄弟姊妹:______名 [5]子女:______名

[6]孫:__________名 [7]伴侶:______名 [8]朋友/同學:______名 [9]家傭:______名

A10. 個人現時每月平均總收入 (港幣)

☐[1] <2000 ☐[2] 2000 - 4999 ☐[3] 5000 - 14999
☐[4] 15000 - 29999 ☐[5] 30000 - 49999 ☐[6] 50000 - 99999
☐[7] ≥100000 ☐[8] 不清楚/不方便回答

家庭每月平均總收入 (港幣) (計算所有同住家庭成員及伴侶。朋友, 同學及家傭除外)

☐[1] <2000 ☐[2] 2000 - 4999 ☐[3] 5000 - 14999
☐[4] 15000 - 29999 ☐[5] 30000 - 49999 ☐[6] 50000 - 99999
☐[7] ≥100000 ☐[8] 不清楚/不方便回答

A11. 請問你於過去 3 年有多常出外旅行或工作?

	從不[1]	每年少於 1 次[2]	每年 1-2 次[3]	每年 3-5 次[4]	每年多過 5 次[5]	差不多每月 1 次[6]
a. 到亞洲地區	☐	☐	☐	☐	☐	☐
b. 到亞洲以外地區（包括俄羅斯及澳洲）	☐	☐	☐	☐	☐	☐

A12. 請問你日常生活中有沒有接觸動物?

☐[1] 經常
- ☐[1] 家中飼養寵物
- ☐[2] 工作會接觸動物（請註明動物種類__________________）
- ☐[3] 其他（請註明地點:_________________ 和動物種類:_________________）

☐[2] 很少

☐[3] 沒有

A13. 如有飼養寵物，家中的寵物數目?

☐[1] 貓：__________ 隻
☐[2] 狗：__________ 隻
☐[3] 鳥：__________ 隻
☐[4] 魚：__________ 條
☐[5] 兔：__________ 隻
☐[6] 龜：__________ 隻
☐[7] 倉鼠：__________ 隻
☐[8] 其他（請註明寵物種類及數目): ______________（____隻）
______________（____隻）

A14. 有沒有吸煙的習慣?

☐[1] 有, 每天吸煙支數? ____________ 支;開始吸煙年歲:___________ 歲

☐[2] 曾經有，但已戒掉（戒掉多久？____________年）

☐[3] 從來沒有

A15. 同住成員有沒有吸煙的習慣?

☐[1] 有, 同住多久? __________年

☐[2] 曾經有，但已分開居住（同住多久? _________年, 分開居住多久? _________年）

☐[3] 從來沒有

A16. 請問你有沒有飲酒習慣?

☐[1] 有

☐[1] 每月飲少過一次（如在特別日子/場合才飲） ☐[2] 每月 1-3 次

☐[3] 每星期 1-3 次 ☐[4] 每星期 4-5 次 ☐[5] 差不多每天

☐[2] 曾經有，但已戒掉（戒掉多久？____________）

☐[3] 從來沒有

A17. 有沒有喝奶茶的習慣?

☐[1] 有（喝了多久？____________年）

如有，有多頻繁?

☐[1] 每月飲少過一次 ☐[2] 每月 1-3 次

☐[3] 每星期 1-3 次 ☐[4] 每星期 4-5 次 ☐[5] 差不多每天

☐[2] 曾經有，但現在沒有（戒掉多久？____________年）

☐[3] 從來沒有

A18. 有沒有喝清茶的習慣? (不包括奶茶)

☐[1] 有（喝了多久？__________年）

a. 有多頻繁?

☐[1] 每月飲少過一次 ☐[2] 每月 1-3 次

☐[3] 每星期 1-3 次 ☐[4] 每星期 4-5 次 ☐[5] 差不多每天

b. 通常喝什麼茶?

☐[1] 紅茶 ☐[2] 綠茶 ☐[3] 不清楚

☐[4] 其他(請註明:__________________)

☐[2] 曾經有，但現在沒有（戒掉多久？__________年）

☐[3] 從來沒有

A19. 有沒有喝咖啡的習慣?

☐[1] 有（喝了多久？__________年）

a. 有多頻繁?

☐[1] 每月飲少過一次 ☐[2] 每月 1-3 次

☐[3] 每星期 1-3 次 ☐[4] 每星期 4-5 次 ☐[5] 差不多每天

b. 通常喝哪一種咖啡?

☐[1] 含咖啡因咖啡 ☐[2] 沒有咖啡因的咖啡 ☐[3] 兩者都喝

☐[2] 曾經有，但現在沒有（戒掉多久？__________）

☐[3] 從來沒有
